# Supplementary material for: Structural basis for recognition and regulation of arenavirus polymerase L by Z protein
Source: Nat Commun. 2021 Jul 5;12:4134. doi: 10.1038/s41467-021-24458-1 (PMC8257661; doi:10.1038/s41467-021-24458-1)
Supplement: Supplementary file 1 — Supplementary Information [file 41467_2021_24458_MOESM1_ESM.pdf]

Supplementary information

Structural basis for recognition and regulation of arenavirus polymerase L by Z protein

H. Kang *et al.*

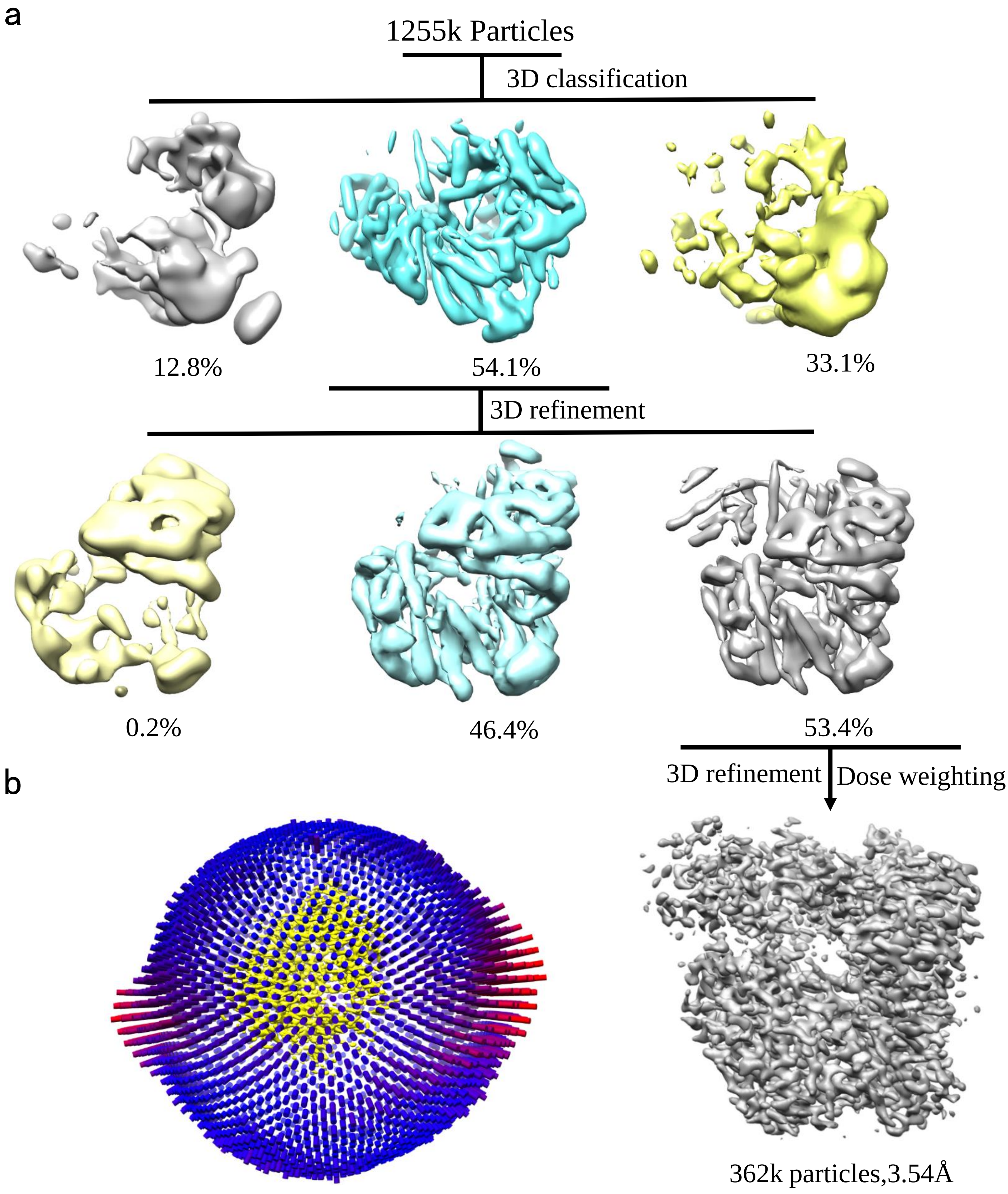
a
1255k Particles
3D classification
12.8%
54.1%
33.1%
3D refinement
0.2%
46.4%
53.4%
3D refinement
Dose weighting
b
362k particles,3.54Å

c

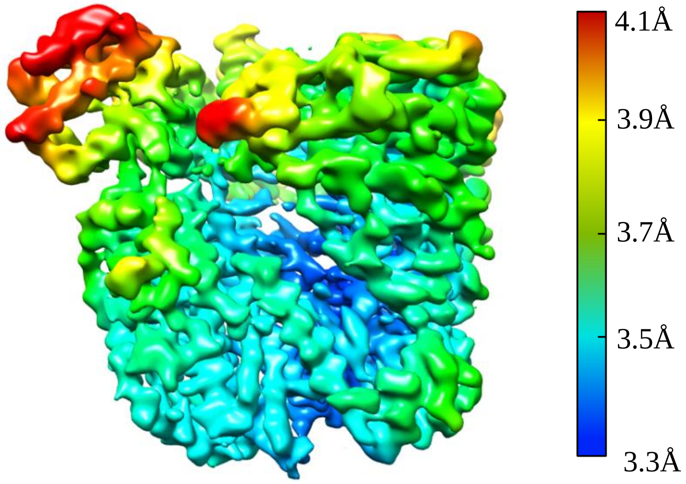
4.1Å
3.9Å
3.7Å
3.5Å
3.3Å

d

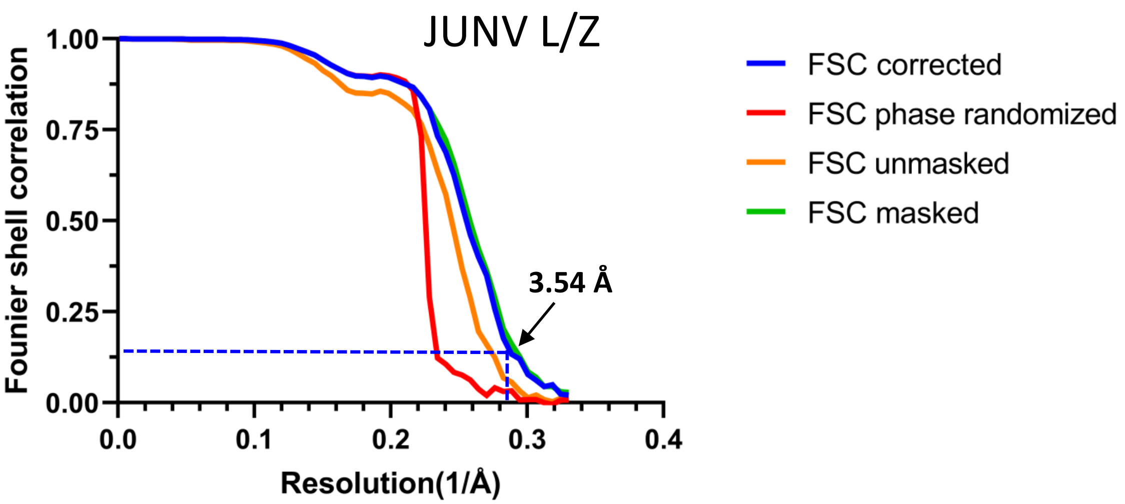
JUNV L/Z
Founier shell correlation
1.00
0.75
0.50
0.25
0.00
0.0
0.1
0.2
0.3
0.4
Resolution(1/Å)
3.54 Å
FSC corrected
FSC phase randomized
FSC unmasked
FSC masked

e

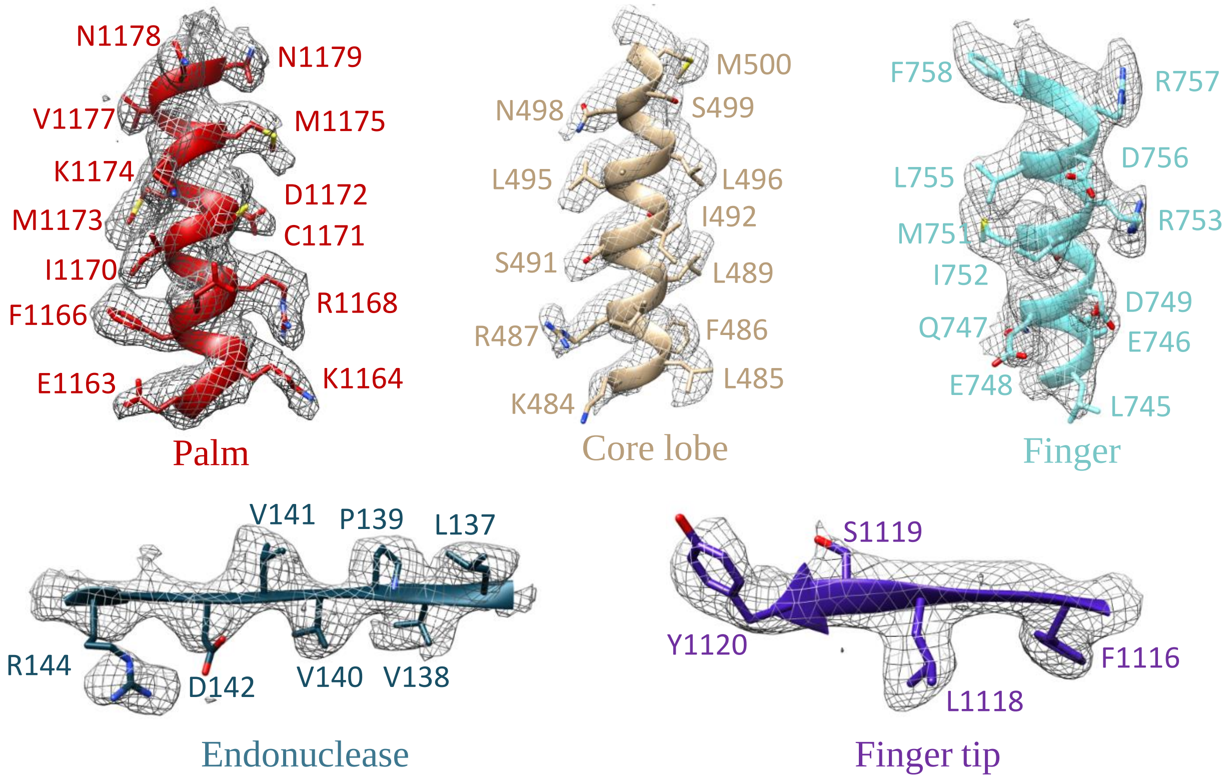
N1178
N1179
V1177
M1175
K1174
D1172
M1173
C1171
I1170
R1168
F1166
E1163
K1164
Palm
M500
N498
S499
L495
L496
I492
S491
L489
R487
F486
K484
L485
Core lobe
F758
R757
D756
L755
R753
M751
I752
D749
Q747
E746
E748
L745
Finger
V141
P139
L137
R144
D142
V140
V138
Endonuclease
S1119
Y1120
F1116
L1118
Finger tip

Supplementary Figure 1: Cryo-EM analysis of the JUNV L–Z complex. a, Brief 3D classification of JUNV L-Z complex particles. We extracted approximate 1255,000 particles, and after two rounds of 3D classification, obtained approximate 362,000 particles for further 3D reconstruction, with the final map reaching a resolution of 3.54Å. **b**, Euler angle distribution of each reconstruction. **c**, Local resolution map for the structure of the JUNV L–Z complex. **d**, The FSC curves for each reconstruction. The FSC 0.143 cut-off values are indicated by blue dashed lines. **e**, Representative density of JUNV L–Z complex reconstructions.

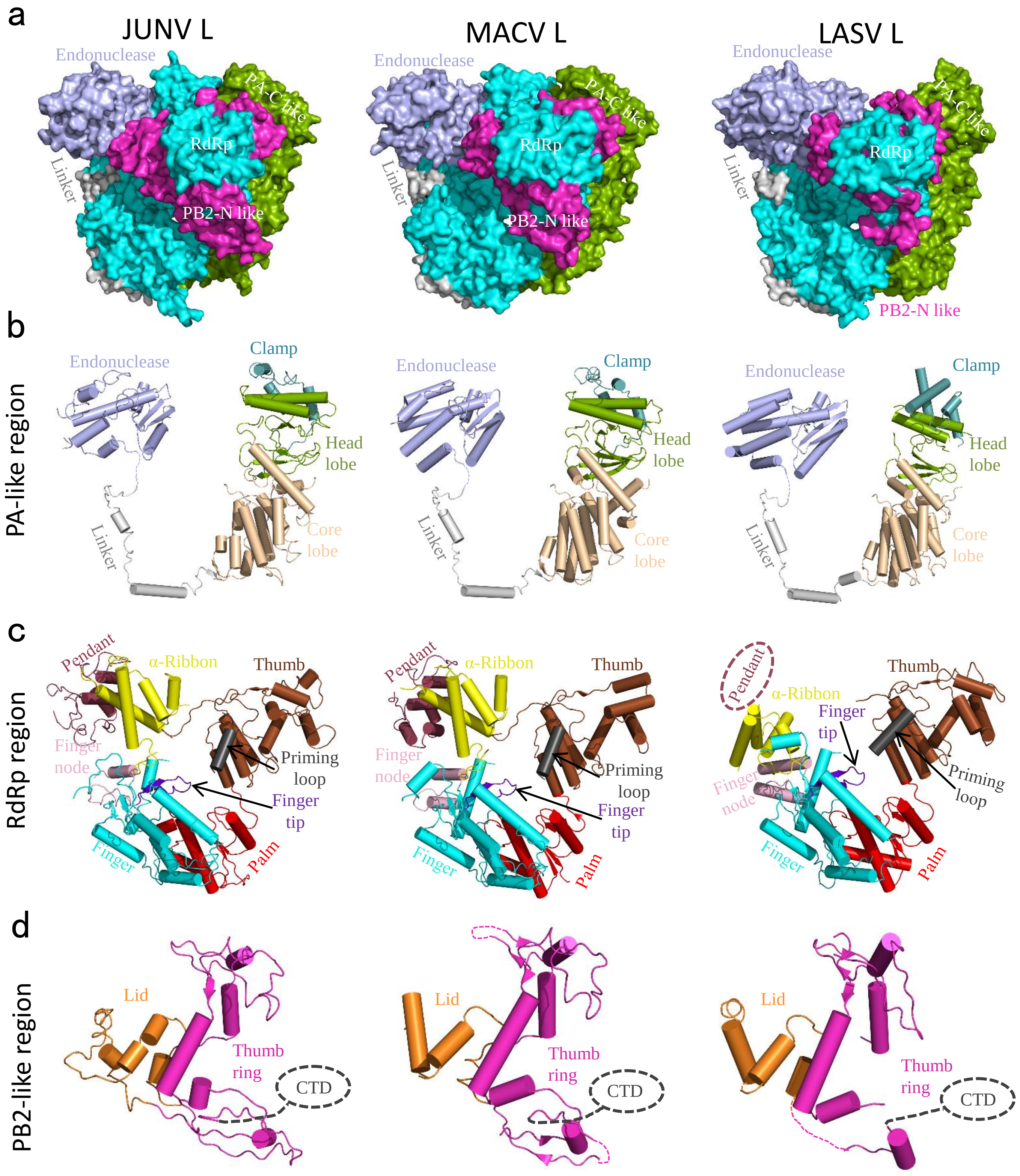

Supplementary Figure 2: Comparison of individual regions of JUNV, MACV (PDB entry: 6KLD) and LASV (PDB entry: 6KLC) polymerases. **a**, Overall architecture of JUNV, MACV and LASV polymerases. **b**, PA-like region of JUNV, MACV and LASV polymerases are shown as cartoon. Each domain is indicated in a unique color. **c**, RdRp regions of JUNV, MACV and LASV polymerases are shown. The pendant domain of LASV L is invisible in the LASV polymerase structure, its putative location is indicated as a raspberry dashed oval. **d**, The CTD of LACV L was found to be truncated during protein expression (PDB entry: 5AMQ). For JUNV and MACV L, the CTD is present in the protein sample but failed to be resolved due to its high flexibility.

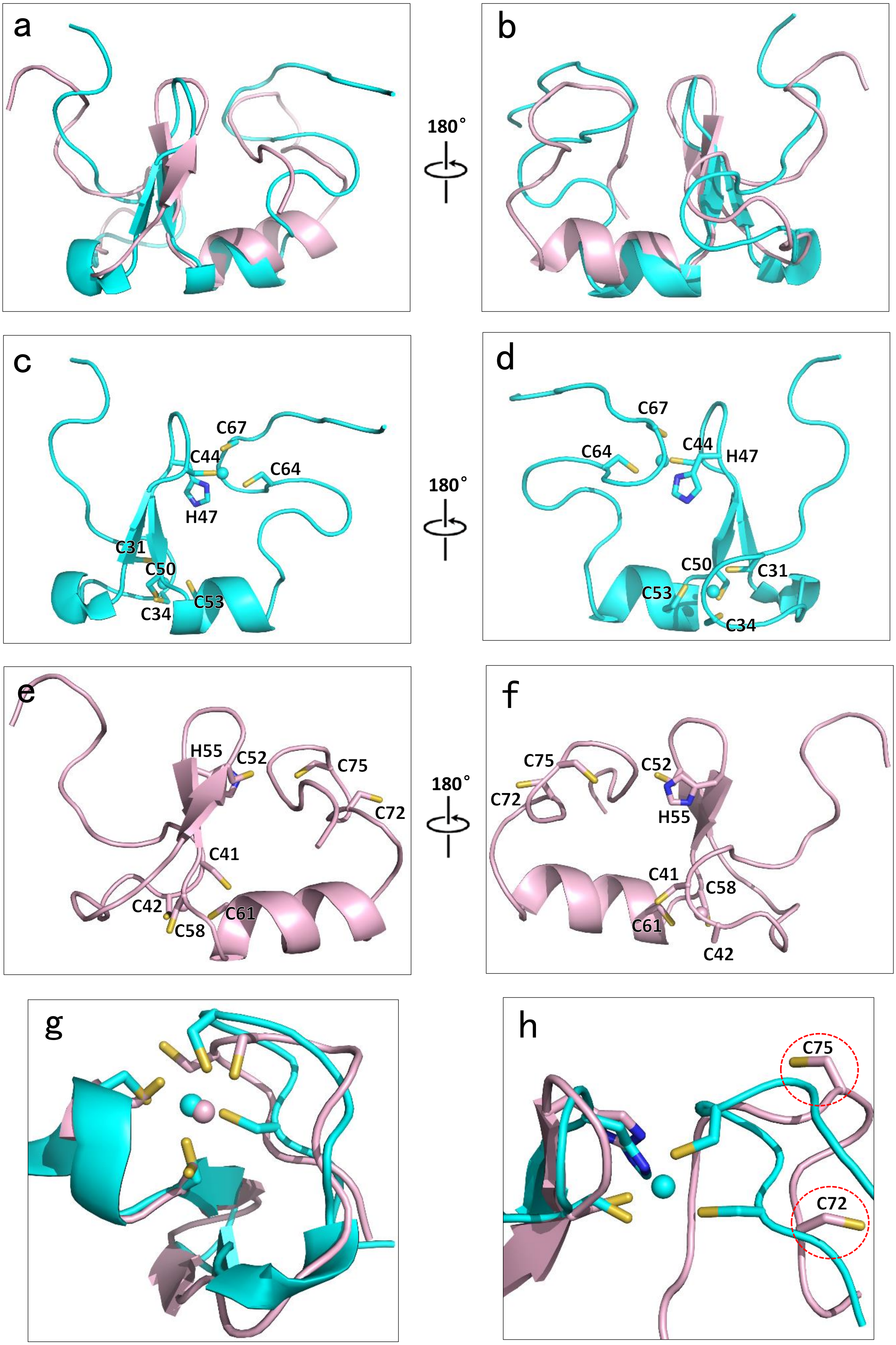
a
b
180°
c
C67
C44
C64
H47
C31
C50
C34
C53
180°
d
C67
C64
C44
H47
C50
C31
C53
C34
e
H55
C52
C75
C72
C41
C42
C61
C58
180°
f
C75
C52
C72
H55
C41
C58
C61
C42
g
h
C75
C72

Supplementary Figure 3: Comparison of Z protein of JUNV and LASV (PDB entry: 2M1S). a,**b**, Alignment of JUNV Z protein with LASV Z protein. The two structures are shown as cartoon, JUNV Z is shown in light pink and LASV Z in cyan. (b) View identical to that shown in (a), but rotated by 180°. **c**,**d**, Z protein of LASV is shown as cartoon. (d) View identical to that shown in (c), but rotated by 180°. **e**,**f**, Z protein of JUNV is shown as cartoon. (e) View identical to that shown in (f), but rotated by 180°. **g**,**h**, Comparison of the zinc finger of JUNV and LASV. JUNV Z is shown in light pink and LASV Z in cyan. In (g) the residues Cys75 and Cys72 of JUNV are too far apart to form a zinc finger. In panels c-g, side chains of residues involved in zinc finger formation are shown as sticks, with oxygen atom shown in yellow, nitrogen atom in blue. Zn atom is shown as a sphere.

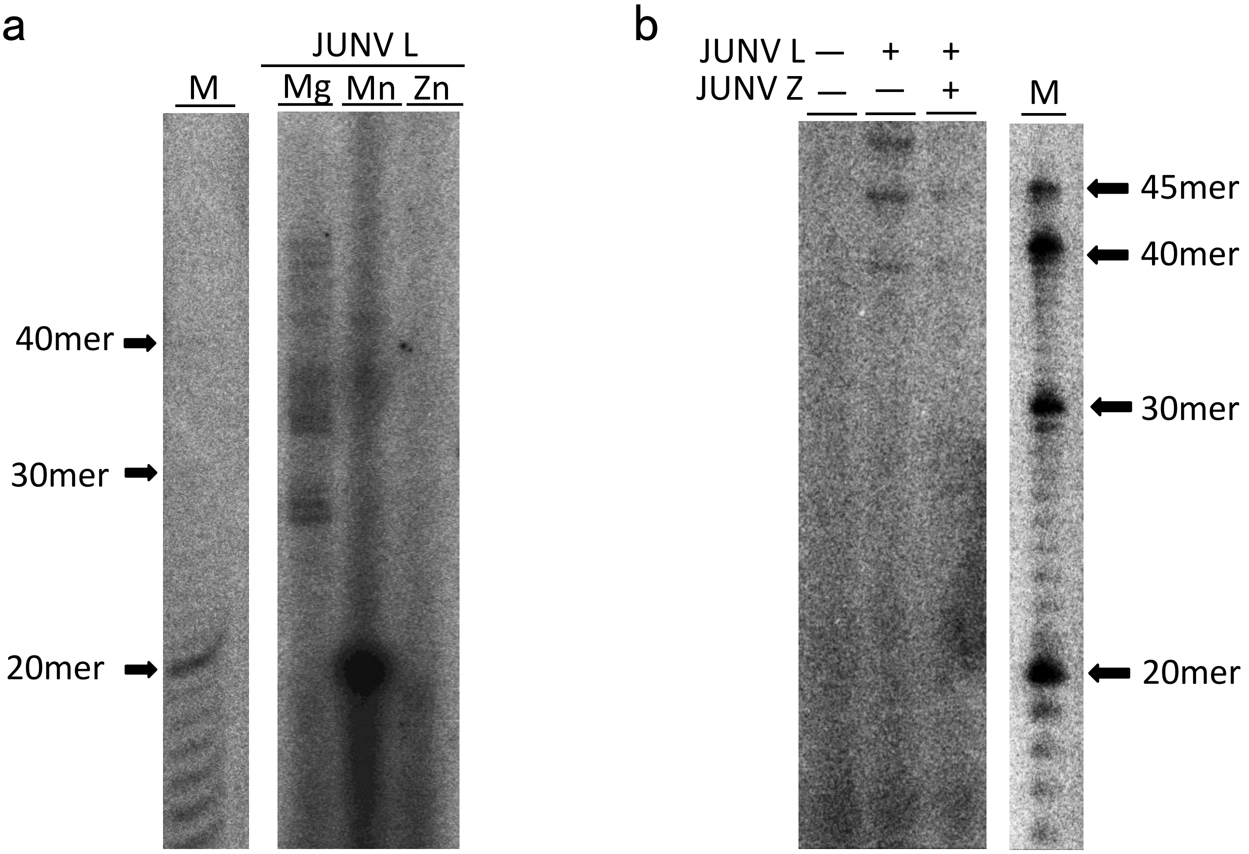

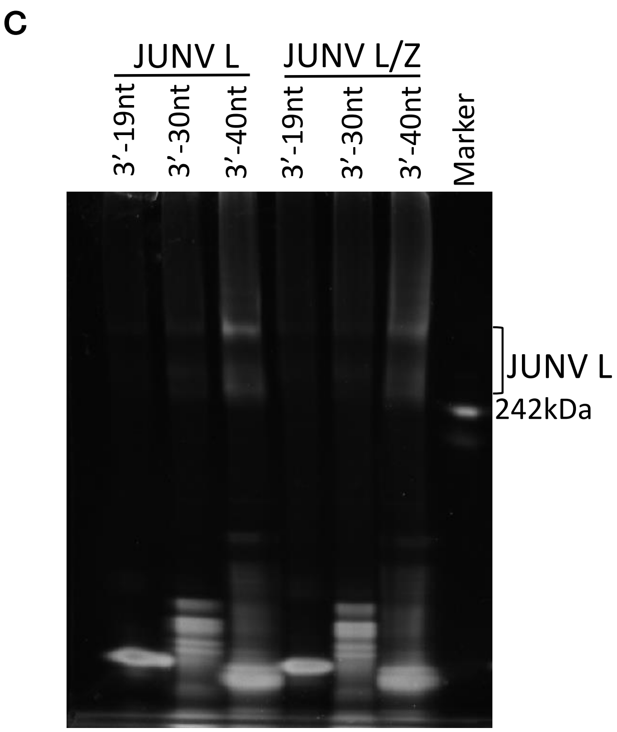

Supplementary Figure 4: *In vitro* functional assays for JUNV L protein or L-Z complex. a, Show the effect of metal ions on enzymatic activity, while Mg^{2+} and Mn^{2+} were found to promote RNA replication activity, while Zn^{2+} can not activate L protein. Final concentration of $Mg^{2+,}$ Mn^{2+} and Zn^{2+} were 5mM, respectively. **b,** Shown the RNA products generated from RNA synthesis reactions supplemented with or without Z. Found that the enzymatic activity of L protein was inhibited in the presence of Z protein. **c**, The affinity between JUNV L protein and vRNA promoter were measured by EMSA. Shown that L can bind vRNA promoter regardless of the presence or absence of Z protein.

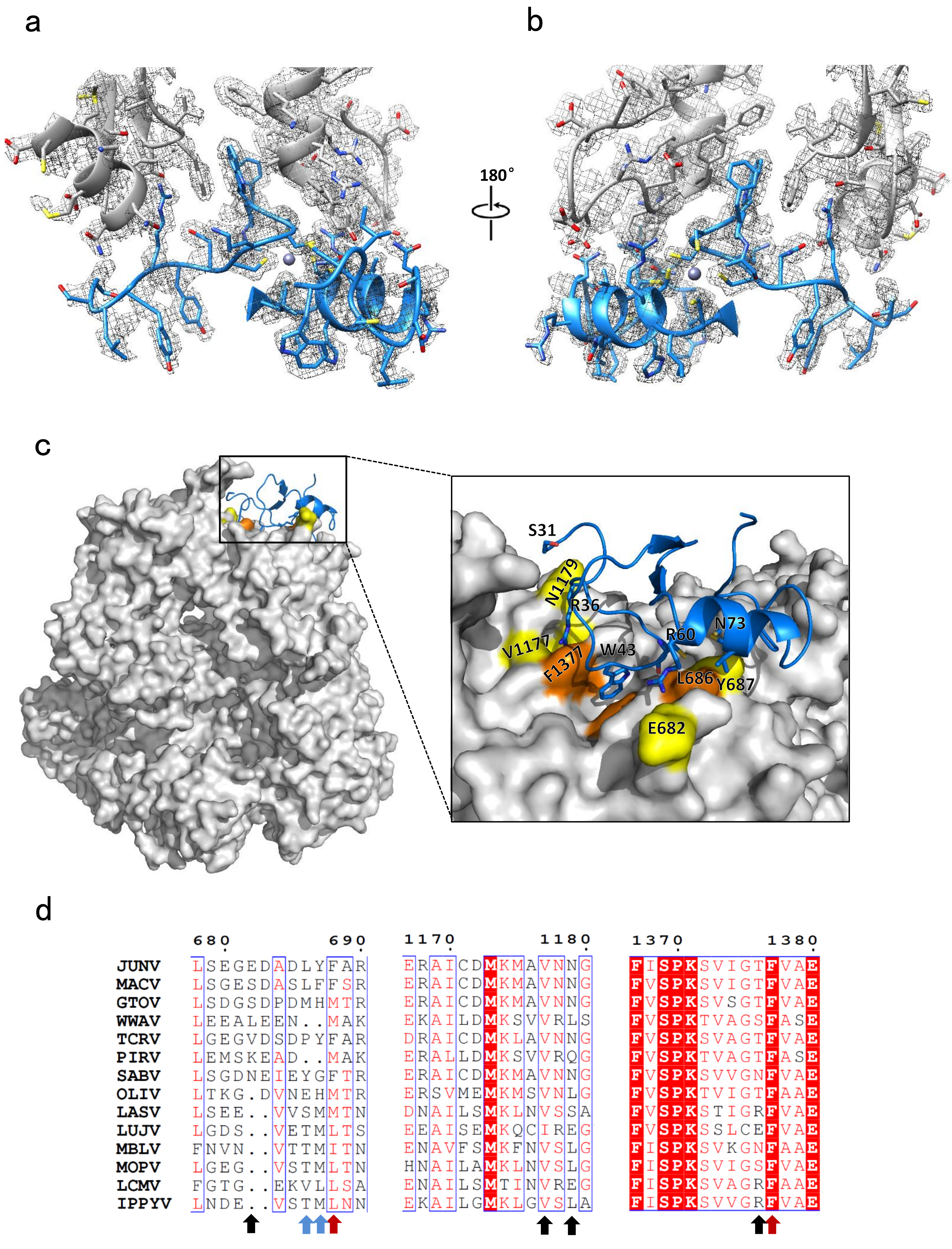
a
b
180°
c
S31
N1179
R36
V1177
F1377
W43
R60
N73
L686
Y687
E682
d
680 690 1170 1180 1370 1380
JUNV LSEGEDADLYFAR ERAICDMKMAVNNG FISPKSVIGTFVAE
MACV LSGESDASLFFSR ERAICDMKMAVNNG FVSPKSVIGTFVAE
GTOV LSDGSDPDMHMTR ERAICDMKMAVNNG FVSPKSVSGTFVAE
WWAV LEEALEEN..MAK EKAILDMKSVVRLS FVSPKTVAGSFASE
TCRV LGEGVDSDPYFAR DRAICDMKLAVNNG FVSPKSVAGTFVAE
PIRV LEMSKEAD..MAK ERALLDMKSVVRQG FVSPKTVAGTFASE
SABV LSGDNEIEYGFTR ERAICDMKMAVNNG FVSPKSVVGNFVAE
OLIV LTKG.DVNEHMTR ERSVMEMKMSVNLG FVSPKTVIGTFAAE
LASV LSEE..VVSMMTN DNAILSMKLNVSSA FVSPKSTIGRFVAE
LUJV LGDS..VETMLTS EEAISEMKQCIREG FVSPKSSLCEFVAE
MBLV FNVN..VTTMITN ENAVFSMKFNVSLG FISPKSVKGNFAAE
MOPV LGEG..VSTMLTN HNAILAMKLNVSLG FISPKSVIGSFVAE
LCMV FGTG..EKVLLSA ENAILSMTINVREG FISPKSVAGRFAAE
IPPYV LNDE..VSTMLNN EKAILGMKLGVSLA FISPKSVVGRFVAE

e

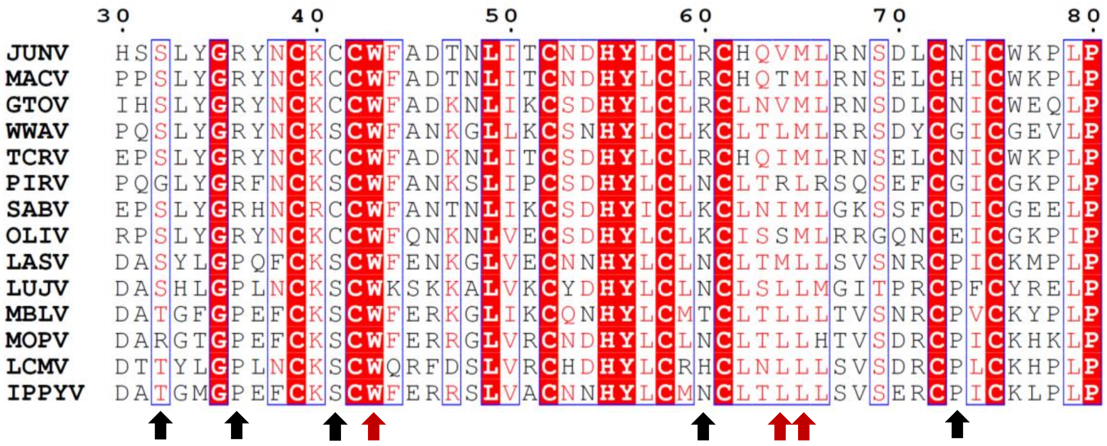

Supplementary Figure 5: Nature of interactions between the JUNV L and Z. a,b, Shown the density map of the interaction interface between L and Z, with L colored in gray and Z colored in blue. All side chains are shown as sticks, with oxygen, nitrogen and sulfur atoms shown in red, blue and yellow, respectively. **c**, Interaction interface between L and Z. L protein is shown in surface presentation, Z protein is shown in cartoon presentation. The box indicate the magnified views of the interactions between L and Z. Side chains of Z involved in hydrogen bonds or hydrophobic interactions are shown as sticks. Residues of L involved in hydrogen bonds are shown in yellow, and those involved in hydrophobic interactions are shown in orange. **d**, Sequence alignment for key residues of L protein involved in the L-Z binding interface, which are highlighted by arrows: black arrows represent the residues involved in hydrogen bonding interaction, red arrows represent the residues involved in hydrophobic interactions, light blue arrows represent the residues involved in both interactions. **e,** Sequence alignment for key residues of Z protein involved in the L-Z binding interface, which are highlighted by arrows: black arrows represent the residues involved in hydrogen bonding interaction, red arrows represent the residues involved in hydrophobic interactions.

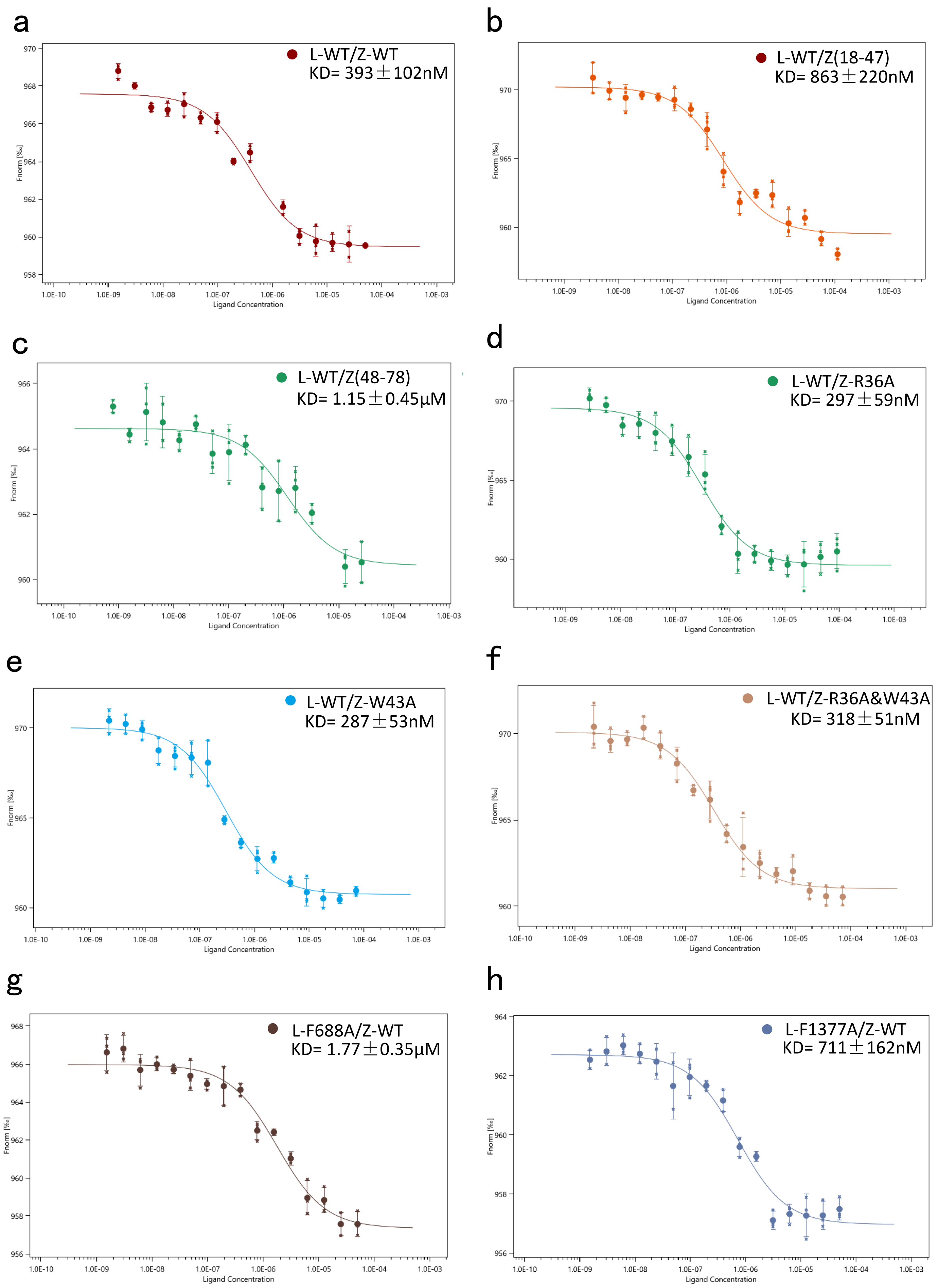
a
L-WT/Z-WT
KD= 393±102nM
970 968 966 964 962 960 958
Fnorm [‰]
1.0E-10 1.0E-09 1.0E-08 1.0E-07 1.0E-06 1.0E-05 1.0E-04 1.0E-03
Ligand Concentration
b
L-WT/Z(18-47)
KD= 863±220nM
970 965 960
Fnorm [‰]
1.0E-09 1.0E-08 1.0E-07 1.0E-06 1.0E-05 1.0E-04 1.0E-03
Ligand Concentration
c
L-WT/Z(48-78)
KD= 1.15±0.45μM
966 964 962 960
Fnorm [‰]
1.0E-10 1.0E-09 1.0E-08 1.0E-07 1.0E-06 1.0E-05 1.0E-04 1.0E-03
Ligand Concentration
d
L-WT/Z-R36A
KD= 297±59nM
970 965 960
Fnorm [‰]
1.0E-09 1.0E-08 1.0E-07 1.0E-06 1.0E-05 1.0E-04 1.0E-03
Ligand Concentration
e
L-WT/Z-W43A
KD= 287±53nM
970 965 960
Fnorm [‰]
1.0E-10 1.0E-09 1.0E-08 1.0E-07 1.0E-06 1.0E-05 1.0E-04 1.0E-03
Ligand Concentration
f
L-WT/Z-R36A&W43A
KD= 318±51nM
970 965 960
Fnorm [‰]
1.0E-10 1.0E-09 1.0E-08 1.0E-07 1.0E-06 1.0E-05 1.0E-04 1.0E-03
Ligand Concentration
g
L-F688A/Z-WT
KD= 1.77±0.35μM
968 966 964 962 960 958 956
Fnorm [‰]
1.0E-10 1.0E-09 1.0E-08 1.0E-07 1.0E-06 1.0E-05 1.0E-04 1.0E-03
Ligand Concentration
h
L-F1377A/Z-WT
KD= 711±162nM
964 962 960 958 956
Fnorm [‰]
1.0E-10 1.0E-09 1.0E-08 1.0E-07 1.0E-06 1.0E-05 1.0E-04 1.0E-03
Ligand Concentration

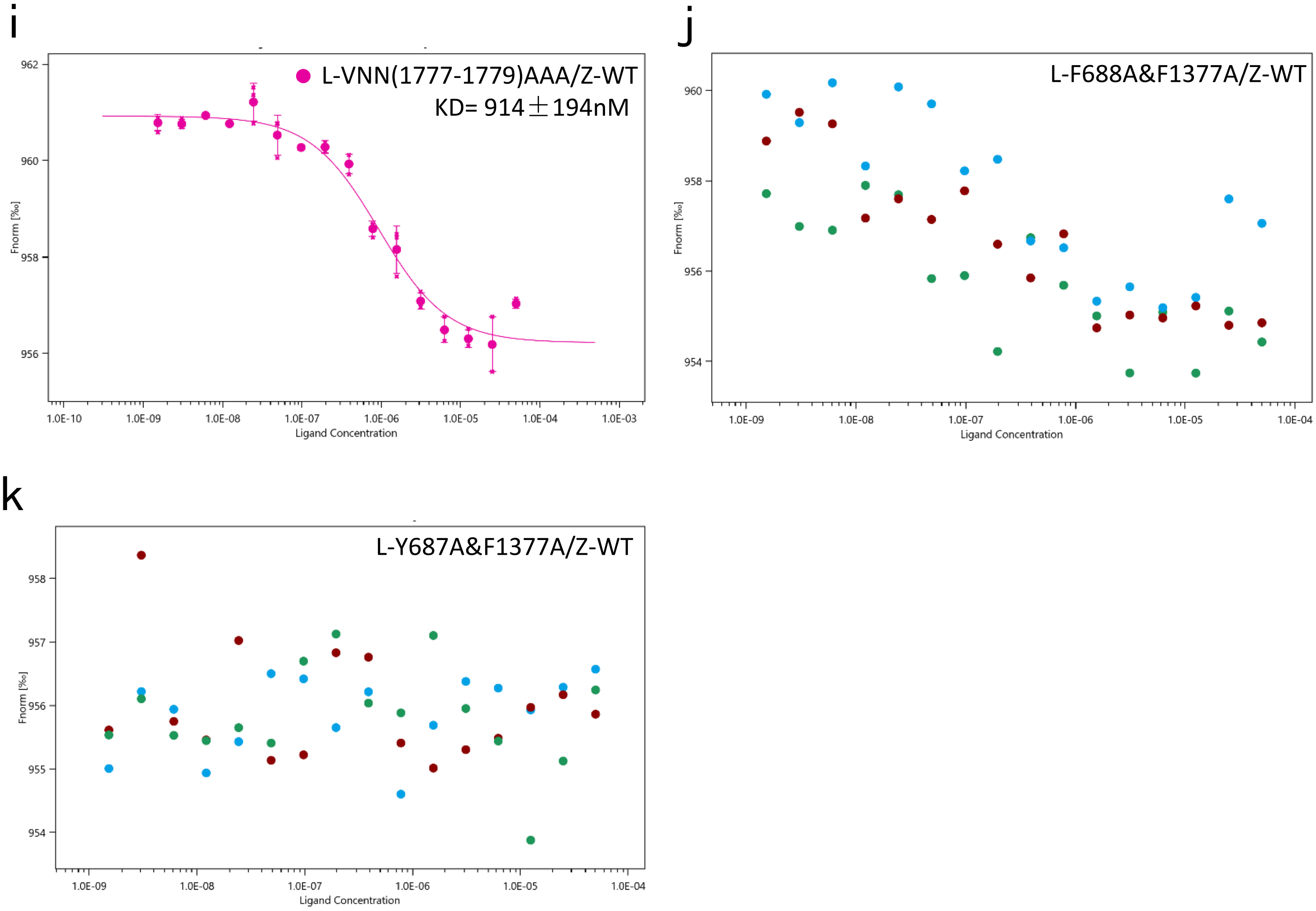

Supplementary Figure 6: The affinity between JUNV L protein (mutants) and Z protein (mutants) were measured using a MST assay. Each MST assay included three groups of parallel experiments, and the average value was taken to fit the combination curve, the error bar represents standard deviation (n = 3). Fluorochrome-labeled wild type L protein and L mutants (F1377A and VNN(1777-1779)AAA) were tested for their binding affinity with full length Z protein, Z mutants and Z peptide (18-47 and 48-78). The normalized fluorescence (1/1000) was plotted for analysis of thermophoresis. **a-k**, MST assay result for interaction of L-WT/Z-WT (a), L-WT/Z(18-47) (b), L-WT/Z(48-78) (c), L-WT/Z-R36A (d), L-WT/Z-W43A (e), L-WT/Z-R36A&W43A (f), L-F688A/Z-WT (g), L-F1377A/Z-WT (h), L-VNN(1777-1779)AAA/Z-WT (i), L-F688A&F1377A/Z-WT (j), L-Y687A&F1377A/Z-WT (k).

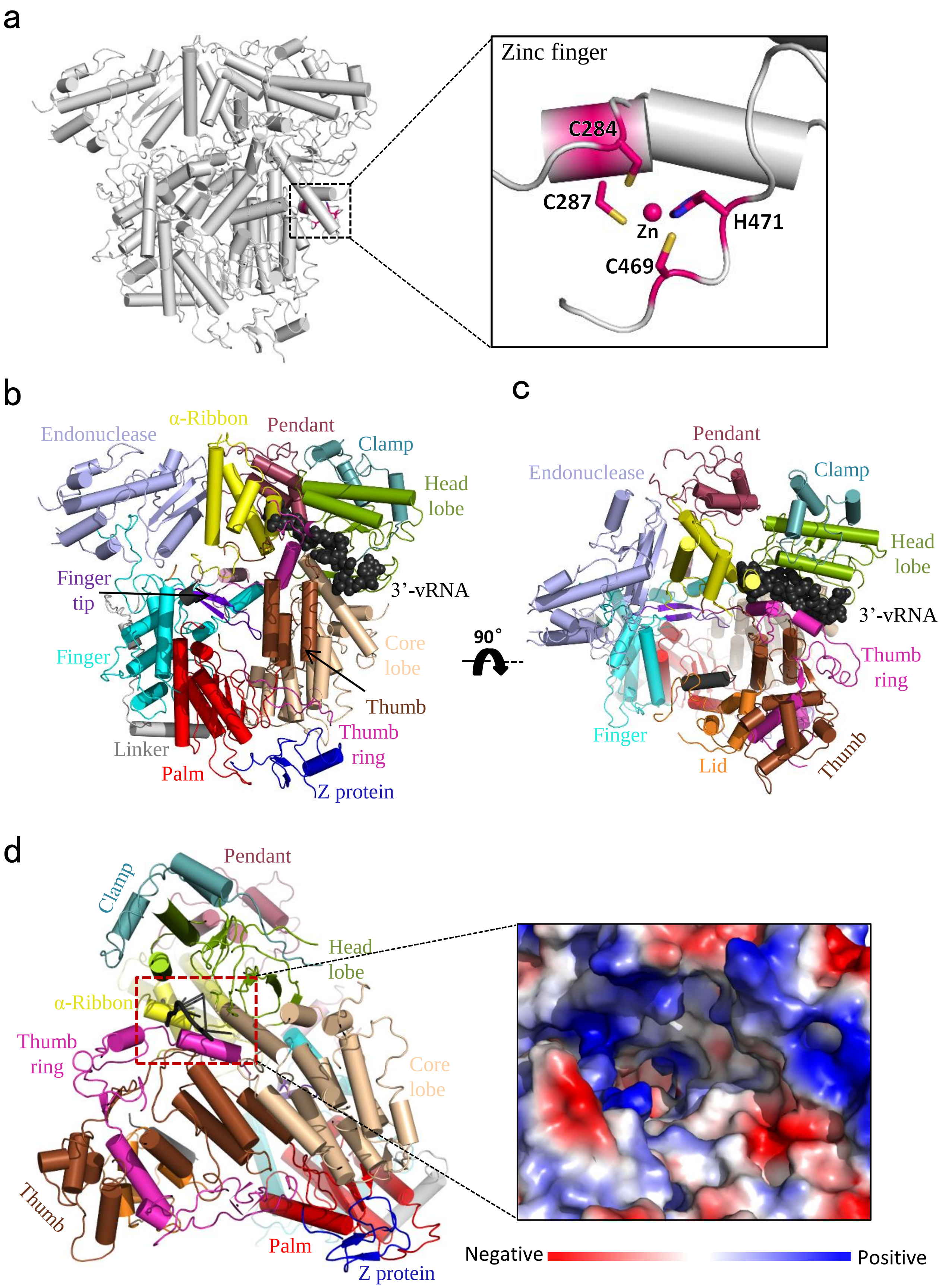
a
Zinc finger
C284
C287
Zn
H471
C469
b
Endonuclease
α-Ribbon
Pendant
Clamp
Head lobe
Finger tip
3'-vRNA
Finger
Core lobe
Thumb
Thumb ring
Linker
Palm
Z protein
c
90°
Pendant
Endonuclease
Clamp
Head lobe
3'-vRNA
Thumb ring
Finger
Lid
Thumb
d
Clamp
Pendant
Head lobe
α-Ribbon
Thumb ring
Core lobe
Thumb
Palm
Z protein
Negative
Positive

Supplementary Figure 7: Analysis of the 3′-vRNA-binding sites of JUNV polymerase. a, Zinc finger of JUNV L protein. Side chains of the zinc finger are shown as sticks and highlighted, the Zn atom involved in zinc finger is shown as hot red sphere. **b**,**c**, Possible 3′-vRNA-binding sites of JUNV polymerases are shown, which are based on the alignment of JUNV L-Z complex structure with MACV L-3’RNA complex structure (PDB entry: 6KLH). The protein structures are shown as cartoon and colored according to the domains. The modelled RNA is shown as a sphere model and colored in black. (b) View identical to that shown in (a), but rotated by 90°. **d**, Electrostatic surface potential representation of the possible 3′-vRNA-binding site in JUNV L protein. The molecular surfaces are colored according to their electrostatic potential from red to blue, negative to positive, respectively.

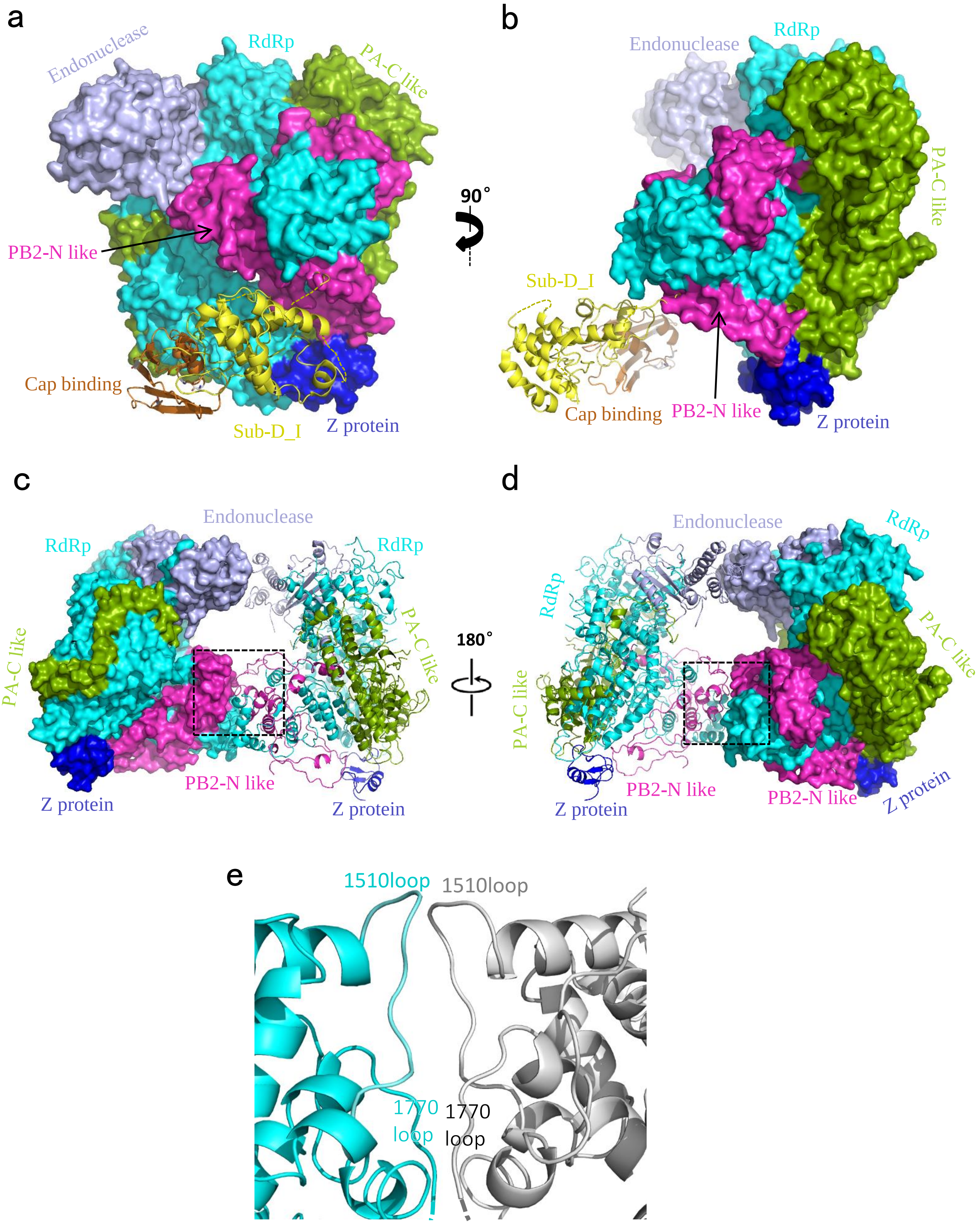
a
Endonuclease
RdRp
PA-C like
PB2-N like
Cap binding
Sub-D_I
Z protein
90°
b
Endonuclease
RdRp
PA-C like
Sub-D_I
Cap binding
PB2-N like
Z protein
c
Endonuclease
RdRp
RdRp
PA-C like
PA-C like
PB2-N like
Z protein
Z protein
180°
d
Endonuclease
RdRp
RdRp
PA-C like
PA-C like
PB2-N like
PB2-N like
Z protein
Z protein
e
1510loop
1510loop
1770 loop
1770 loop

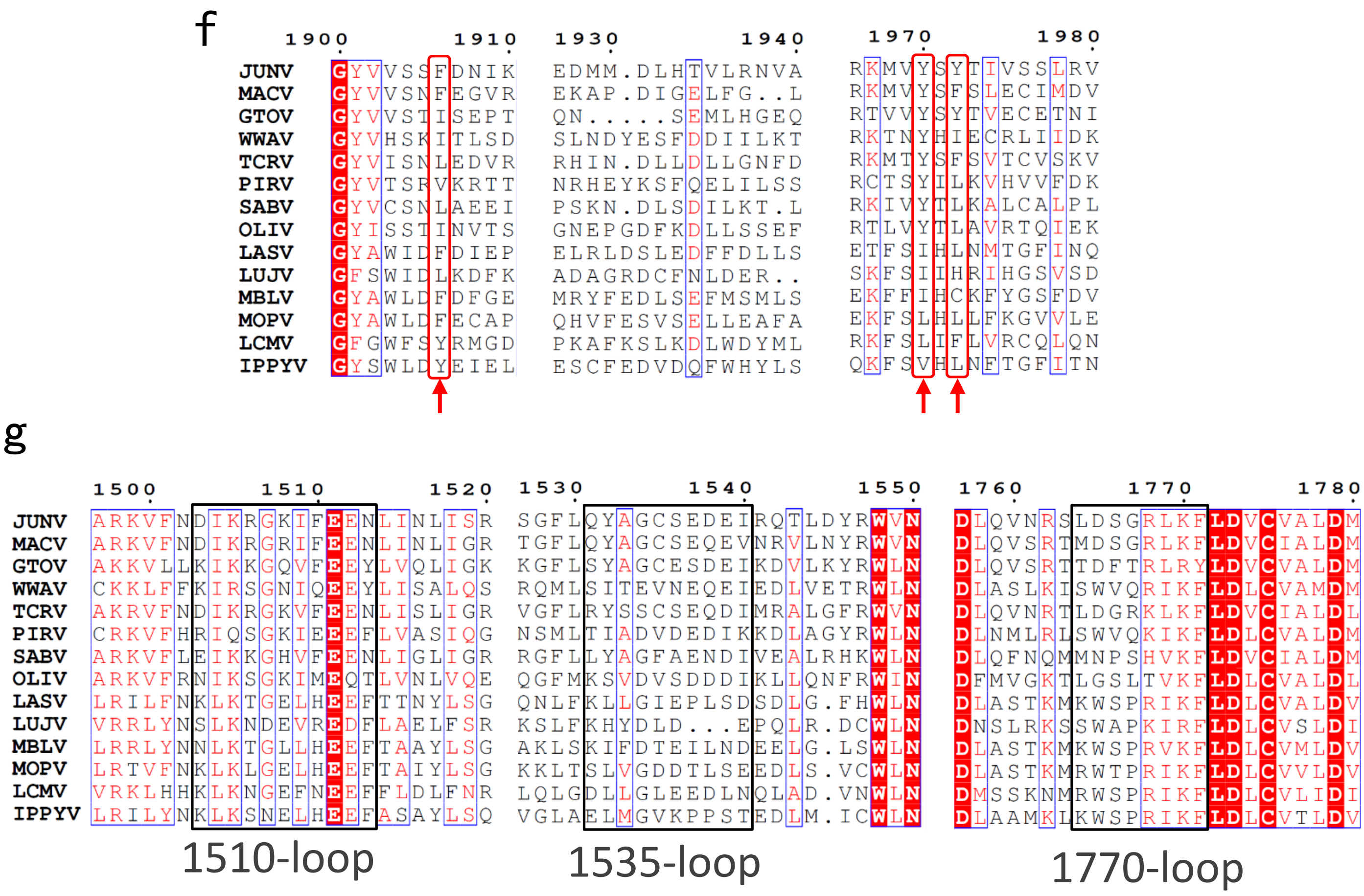

Supplementary Figure 8: Modelling of the JUNV L homodimer and the JUNV L C-terminal domain. a,**b**, Putative location of JUNV polymerase C-terminal domain, which is based on the alignment of JUNV L-Z complex structure with MACV polymerases homodimer structure (PDB entry: 6KLH). JUNV L-Z complex is shown as surface and colored by domains. The modelled CTD is shown as cartoon, and was divided into two parts: caping domain and sub-D_I. (b) Same view as in (a), but rotated by 90°. **c**,**d**, Structural model of the JUNV L-Z complex homodimer. The model is based on the MACV polymerase homodimer structure (PDB entry: 6KLH). The two JUNV L-Z complex proteins are colored by domains, one shown as surface, one shown as cartoon. (d) Same view as in (c), but rotated by 90°. **e**, Close-up view of the predicted dimeric interface. Key contacting loops are highlighted. **f**, Sequence alignment for three key residues potentially involved in cap binding are highlighted by red boxes and marked with arrows. The three residues were identified by comparing the structure of JUNV L-Z complex with the structure of MACV polymerase homodimer. **g**, Sequence alignment for key regions of the dimeric interface, with three key motifs highlighted by black boxes: loop 1510, loop 1770 and loop 1535. The loop 1510 and loop 1770 are involved in the formation of dimeric interface, the loop 1535 is involved in stabilizing the C-terminal function.

**Data collection**	
EM equipment	FEI Titan Krios
Voltage (kV)	300
Detector	Gatan K2
Pixel size (Å/pixel)	0.65
Electron dose (e^-/Å^2)	1.2
Defocus range (μm)	1.2-2.5
**Reconstruction**	
Software	Relion3.0
Number of used particles	362,000
Symmetry	C1
Map sharpening B-factor (Å^2)	-157.3
Final resolution (Å)	3.54
**Model building**	
Software	Coot
**Model Refinement**	
Software	Relion3.0, Phenix
Map CC (mask)	0.776
Map CC (peaks)	0.639
Map CC (volume)	0.730
Mean CC for ligands	0.715
B factors (Å^2)	
Protein	34.8
ligands	49.1
R.M.S. deviations	
bonds (Å)	0.0095
angles (º)	1.51
**Model composition**	
L Protein residues	1648
Z protein residues	52
Zn^{2+}	2
Mg^{2+}	1
**Validation**	
MolProbity score	1.99
Clash score	4.82
Ramachandran plot	
Outliers (%)	0.0
Allowed (%)	14.0
Favored (%)	86.0
Rotamer outliers (%)	1.15
Cβ outliers (%)	0

Supplementary Table 1: Statistics of Data Collection, Image Processing and Model Building.

Subunit Name	Chain	Total residues/ range built	Poly-ALA model	Unmodelled residues	% atomic model	Co-factors
L	A	2210/<br>1-173,<br>179-195,<br>200-302,<br>320-460,<br>466-511,<br>520-803,<br>820-876,<br>884-921,<br>942-1042,<br>1085-1251,<br>1265-1567,<br>1577-1590,<br>1608-1758,<br>1765-1817	-	174-178,<br>196-199,<br>303-319,<br>461-465,<br>512-519,<br>804-819,<br>877-883,<br>922-941,<br>1043-1084,<br>1252-1264,<br>1568-1576,<br>1591-1607,<br>1759-1764,<br>1819-2210	1648/2210	Mg^{2+}, Zn^{2+}
Z	B	94/31-82	-	1-30,83-94	52/94	Zn^{2+}

Supplementary Table 2: Summary of the model.

a, The DNA sequence of the codon-optimized JUNV L gene:

ATGGAAGAGTCTGTGAACGAAATCAAGACACTGATCCGCAAGCACTTCCCCGAGCGCC
AGGAATTGGCTTACCAGAGAGACATCTTCTTGAGCCAGCACCACCCTAGCTCCTTGTTG
CTAGAGGGATTCAAGCTGTTGAGCAGCCTGGTGGAATTGGAAAGCTGCGAGGCCCACG
CTTGCCAGATCAACTCTGACCAGAAGTTCGTGGACGTCATCCTGTCAGACCACGGTATC
TTGTGCCCTACATTGCCAAAGGTCATCCCAGACGGTTTCAAGCTCACCGGCAAGACATT
GATCTTGTTGGAAACCTTCGTGAGAGTCAACCCCGACGAATTTGAGCGCAAGTGGAAG
TCAGACATGTCTAAGTTGTTGAACTTGAAGTCTGACCTGCTGAGAGTCGGTATCACTCT
GGTGCCCGTCGTGGACGGTAGAAGTAGCTACTCTAACAGATTCCTCGCTGACTGGGTCG
TCGAGCGCGTGCGCTGGCTGCTGATCGACATCTTGAAGAAGTCTAAGTTCATGCAGGAA
ATCAACATCGAAGAACAAGAATACCAGAGATTGATCCACTCATTGTCCAACACAAAGA
ACCAGTCACTGGGATTGGAAAACATCGAATGCCTGAAGAAGAACTCTTTGGGTTACGA
CGAGCGCCTGAACGAGTCATTGTTCGTCGGAGTGAGGGGCGACATCCGCGAGTCTGTG
ATCAGGGAAGAGCTAATCAAGCTCAGATTCTGGTTCAAGAAGGAAATCTTCGACAAGC
AGTTGGGAAAGTTCAAGTTCTCACAGAAGTCCAACTTGATCAACGACTTGATCTTGCTC
GGTAGCCACAAGGACTCTGACGTGCCTAGCTGCCCTTTCTGCGCTAACAAGCTGATGGA
CGTCGTGTACTCCATCGCTTTGCACCCAATCGACGAAGTGAACATGGAGCGCCAGTCCG
ACGAAAACTCTACCTCCATCGACGCCGTCGAACGTTGCTACTTGCAGGCTTTGTCAGTC
TGCAACAAGGTGAAGGGCCTGAAGGTGTTCAACACCCGTAGAAACACACTGCTGTTCC
TCGACCTCGTGTTGCTCAACTTGCTGTGCGACCTGTTCAAGACATACGACGGCGCTATC
GTGAGACTGCGTAACGCCGGTATCGTCGTGGGCCAGATGCTCATGCTGGTGAACGACCG
TTTGTTGGACATCTTGGAGGCTATCAAGCTGATCCGTAAGAAGTTGATGACTTCCCCAA
AGTGGGTCCAGATGTGCTCACGCACTTTGAAGAACAGCCACCAGGACCTCTGGTTGCA
GTTGGAAAAGCTCATCAAGCACCCCGACATGGACAACCTCATGATCCTCGCCCAGGTGT
TGGTCTCTGACCGCCCCGTGATGCGTTACACAATCGACCGTGAGTTCGAGAAAATCTGC
CGCCACCAGCCATTCTCTAGCCTCGTCGAGGGCGAGCAGAAGAAGTTGTTCCGCATCCT
CTCCTCTATCAGCCTGGCTTTGGTCAACAGCATGAAAACCTCTTTCTCCTCACGCTTGTT
GATCAACGAACGCGAATACTCACGTTACTTCGGTAACGTGCGCCTGCGCGAGTGCTATG
TTCAGCGTTTCCACCTGACAAAGAACACCTTCGGCTTGCTGTTCTACCAAAAAACCGG
AGAGAAGTCTAGGTGCTACAGCATCTACTTGTCTACAAACGGAGTGTTAGAGGAACAG
GGTAGCTTCTACTGCGACCCAAAGCGTTTCTTCCTCCCAATCTTCTCCGAGGACGTGCT
GATCGAAATGTGCGAGGAAATGACAAGCTGGTTGGACTTCAGCCACGAATTGATGACT
ATGACTCGCCCAATCTTGCGTCTGTTGGTCCTCGCTGTGTTGTGCAGCCCTAGCAAGCG
TAACCAGACATTCTTGCAGGGTTTGCGTTACTTCCTGATGGCTTACGCTAACCAGATCCA
CCACGTTGACTTGATGTCAAAGCTGAGAGTGGACTGCATGTCCGGTAGCGAGGTGCTC
ATCCAGCGTATGGCCGTCGAATTGTTCCAGATCATCCTGTCCGAGGGAGAGGACGCTGA
CCTGTACTTCGCCCGCCGTTTCAAGTACTTGCTGAACGTGTCTTACTTGTGCCACCTCGT
CACAAAGGAAACCCCCGACCGCCTCACTGACCAGATCAAGTGCTTCGAGAAGTTCGTC
GAGCCAAAGGTGAAGTTCGGATGCGTGGTCGTCAACCCACCCTTGAACGGCAGCCTGA
CATTGGAGCAAGAAGATACTATGATCCGCGGTTTGGACCGCTTCTTCTCTAAAGAGGCT
AAGACCTCTAGCGACACACAGATCCCTGGAGTGTGCAAGGAAATCCTGTCCTTCTGCAT
CTCATTGTTCAACCGCGGCAGGCTGAAGGTCACCGGCGAATTGAAGTCCAACCCATACC
GCCCAAACATCACCTCTACCGCCCTGGACCTGTCTAGCAACAAGAGCGTTGTTATCCCA
AAGCTCGACGAATTGGGTAACATCCTGTCAGTGTACGACAAGGAAAAGCTCGTGAGCA
CATGCGTGAGCACTATGGCCGAGCGTTTCAAGACTAAGGGCCGTTACAACCTGGACCCC

GACAGCATGGACTACCTCATCTTGAAGAACCTCACCGGATTGGTCAGCACCGGCAGCCG
CACTAGAACTAACCAGGAAGAATTGTCAGTGATGTACGAATCTTTGACAGAGGACCAGG
TCCGCGCCTTCGAGGGTATCCGTAACGACGTGCAGATGACCCTCGCTAAGATGGCTAAC
TCCGAGGGATCTAAGGTGGAAACTAGCAAGTTGAAGTCTAAGAACTTGTCTGTGGACG
AGAGGGAGTCACTCGAATTGTTGTGGGCCCCATTCGGAGTGATGCGCGAAATCAAGGCC
GAGGTGTCCATGCACGAGGTGAAGGACTTCGACCCCGACGTGTTCCGCTCAGACGTGT
ACAAGGAATTGTGCGACACAGTGTACCTCAGCCCCTACAAGCTGACCTACTTCCTCGAA
GCCCCACAGGACATCTGCCCTTTGGGATTGCTGCTGAAGAACCTCACTACAATCGCTTA
CCAGGAAGAAGAGTTCTTTGAGTGCTTCAAGTACCTGTTGATCCAGGGCCACTACGACC
AGAAGCTCGGATCATACGAGCACCGCTCTAGGTCCCGTTTGGGATTCTCTAGCGAGGTG
TTGAAGCTGAAGGACGAGGTGAGACTGAGCACTAGGGAATCCAACTCCGAGGCTATCG
CTGACAAGCTGGACAAGTCCTACTTCACAAACGCCGCTCTGCGCAACCTGTGCTTCTAC
TCTGACGACTCCCCAACCGAGTTTACCAGCATCAGCTCCAACACAGGCAACTTGAAGTT
CGGATTGTCATACAAGGAACAGGTCGGTAGCAACCGCGAATTGTATGTCGGCGACTTGA
ACACTAAGCTCATGACACGCCTGGTGGAAGATTTCAGCGAGGCCGTCGGCAGCTCCATG
AGATACACTTGCCTGAACTCCGAAAAGGAATTTGAGAGAGCTATCTGCGACATGAAGAT
GGCTGTGAACAACGGCGACTTGTCATGCAGCTACGACCACTCTAAGTGGGGCCCTACAA
TGTCTCCCGCCCTGTTCCTGTCCTTCCTGCACACTCTGGAATTGAAGAACCCTAGAGAC
CGCACTAAGGTGAACCTGGAGCCCGTCATGAACATCCTCAAGTGGCACCTGCACAAGG
TCGTGGAGGTCCCAATCAACGTGGCCGAGGCTTACTGCGTCGGCAAACTCAAGCGTAGC
CTCGGCCTCATGGGATGCGACTGCACCAGCGTCGGAGAGGAATTTTTCCACCAGTACCT
TCAGTCTCGTGACCAGGTCCCTTCTCACATCATGAGCGTGCTGGACATGGGCCAGGGTA
TCCTGCACAACACCTCAGACTTGTACGGTCTGATCACCGAGCAGTTCCTGTGCTACGCC
TTGGACCTCCTGTACGACGTCATCCCCGTGACTTACACATCTAGCGACGACCAGGTGTCT
TTGATCAAGATCCCTTGCCTGTCCGACGAAAAGTGCCAGGACCGTACCGAACTGTTGGA
AATGGTGTGCTTCCACGAGTTCCTGTCCTCTAAGTTGAACAAGTTCATCTCCCCTAAGTC
AGTGATCGGCACATTCGTGGCCGAGTTCAAGTCACGTTTCTTCGTCATGGGAGAGGAAA
CCCCATTGCTGACAAAGTTCGTCAGCGCCGCCCTCCACAACGTGAAGTGCAAGACCCCT
ACACAGTTGTCCGAGACTATCGACACTATCTGCGACCAGTGCATCGCTAACGGAGTGTC
TACCCACATCGTGTCAAAGATCAGCATCAGAGTGAACCAGCTGATCCGTTACTCCGGATA
CAGAGAAACCCCATTCGGCGCTATCGAGGAACAGGACGTCAAGGACTGGGTGGACGGT
AGCCGCGGATACCGTTTGCAGCGTAAGATCGAGGCTATCTTCTCAGACGACAAGGAAAC
AATGTTCATCCGTAACTGCGCCCGTAAGGTGTTCAACGACATCAAGAGAGGCAAGATAT
TTGAAGAAAACCTGATCAACTTGATCTCCCGCGGCGGAGACGAGGCTTTGTCCGGTTTC
TTGCAGTACGCCGGATGCAGCGAAGATGAAATCAGGCAGACTCTGGACTACCGTTGGGT
GAACCTCGCATCCTTCGGCGACTTGAGATTGGTGTTGCGTACTAAGCTCATGACTAGCA
GGCGCGTCCTCGAAAAAGAAGAAATGCCTACATTGATCAAGACTATCCAGTCCCGTTTG
TCCCGTAACTTCACTAAGGGCGTCAAGAAGATCCTCGCTGAGTCTATCAACAAGTCAGC
TTTCCAGTCTAGCGTCGCTAGTGGATTCATCGGATTCTGCAAGTCTATGGGTAGCAAGTG
CGTCCGCGACGGCAAAGGCGGTTTCCTGTACATCAAGGACATCTTCACCCGTATCATCCC
ATGCCTGTGCGGTATCTGCGAGCGTAAGCCAAAGGTCATCTACTGCCAGAAGTCTTTGC
AAGAGGTCAACCAGTTCTCTAAGCCTATCTTGTGGGACTACTTCTCATTGGTCCTCACTA
ACGCCTGCGAACTGGGCGAATGGGTGTTCTCTGCCGTGAAGTCTCCCCAGGCCCCCCTG
GTGTTGTGCAACAAGAACTTCTTCTGGGCCGTGAAGCCTAAGGCCGTCCGCCAGATCGA
GGATCAGTTGGGTATGAACCATGTTTTGCACTCTATCCGTAGAAACTACCCTAAGCTGTT
TGAAGAGCACCTCGCCCCATTCATGAACGACCTTCAGGTGAACCGTAGCCTCGACTCCG

```
GCAGGCTGAAGTTCCTGGACGTGTGCGTCGCTTTGGACATGATGAACGAAAACTTGGGT
ATCATCAGCCACCTCCTCAAGGTGAGAGACAACAACGTGTACATCGTGAAGCAGTCTGA
CTGCGCATCTGCCCACGTCAGGCAGTCCGAATACACCAACTGGGAGGTCGGTATCAGCC
CACAGCAGGTGTGCCGTAACTTCATGGTGCAGGTGGTGTTATCATCCATGATCAACCCCC
TCGTGATGTCTACCTCCTGCTTGAAGTCTTTCTTCTGGTTCAACGAGGTCCTGGACTTAG
AGGACGACAGCCAGGTGGACCTGGCTGAGCTAACAGACTTCACTCTGTCTATCAAGAA
CAACAAGGTGAGCAGGGCTATGTTCGTCGAGGACATCGCTATGGGATACGTTGTCTCTA
GCTTCGACAACATCAAGGTGTTCCTGGAATCCGTCTCTGTGGACAACATCTCACTGCTC
CCCCAAGAGGACATGATGGACCTGCACACAGTGCTCCGTAACGTCGCTTGCCAAGAGG
CCGTGAAGCTCAAGCTGATCATCCAGGTCGAGCACACCCGCGTCAGCACTAAGTTCAA
GTTGCGTAGAAAGATGGTCTACTCTTACACAATCGTATCCTCCCTCCGCGTTGATGACGT
CTCTACCCCCGAATTGGAATTGAACGTGGACACTATGTCTCAGTGCGTGAGCGGTAGCG
AGGGTAACCACTCATTGCTGGACGGCGCCTTGGTCATCGCTTCCTTGCCTTTGTTCACCG
GCCACGAATCTTTCGACTTGGCCGGTTTGTTCATCGACGCCGGTTACGCTTTGACTAACG
ACGACAACATCTTGGGTCACGTCAAGTTCAACTTCGGAGACTTCTACTCCGAAATCTCT
AACAAGTACGCTTACGACTTGATCGGTCCAGACAACCTCGGCGAGCCATTGGTGTTGAA
GGAAGGTGTGTTCTACAGGGGCAACGAAAGGCTGTCCACTTACAGAGTGGAATTGAGC
GGAGACGTCATCGTGAAGGCTATCGGCGCTTTGGAGGACATCGACTCAGTGGAAACTTT
GCTCTCACAGTTGTGGCCATACCTCAAGATGACCTCCCAGACTATCTTGTTCCAGCAGG
AAGATTTCGTGCTCATCTACGACTTGCACAAAGAGCAGTTGATCCGCTCCCTGGACAAG
TTCGGCGACTGGCTCGAATTTTCTAACTTCAAGGTCGCTTTCTCTCGTAGCCTCAACGAC
TTGCTGATCTCTGACCCCCAGGGTCAGTTCAGGCTGAAGGGAGTGACCTGCCGCCCTTT
GAAGCACAAGGTCGAAATCAAGGACATCGACTAA
```

b, The DNA sequence of the codon-optimized JUNV Z gene:

```
ATGGGAAACTGCAACGGCGCGTCGAAGTCCAACCAGCCAGACAGCTCCCGCGTGACCC
AGCCCGCCGCCGAGTTCAGAAGAGTCGCTCACAGCTCCTTGTACGGTAGATACAACTGC
AAGTGCTGCTGGTTCGCTGACACCAACCTGATCACCTGCAACGACCACTACTTGTGCTT
GAGATGCCACCAGGTCATGTTGCGTAACTCCGACTTGTGCAACATCTGCTGGAAGCCATT
GCCTACCACCATCACCGTCCCTGTCGAGCCTACCGCCCCTCCTCCACTAA
```

Supplementary Table 3: a, The DNA sequence of the codon-optimized JUNV L gene. b, The DNA sequence of the codon-optimized JUNV Z gene.
