# Supplementary figures and images for: Structural basis for recognition and regulation of arenavirus polymerase L by Z protein
Source: Nat Commun. 2021 Jul 5;12:4134. doi: 10.1038/s41467-021-24458-1 (PMC8257661; doi:10.1038/s41467-021-24458-1)

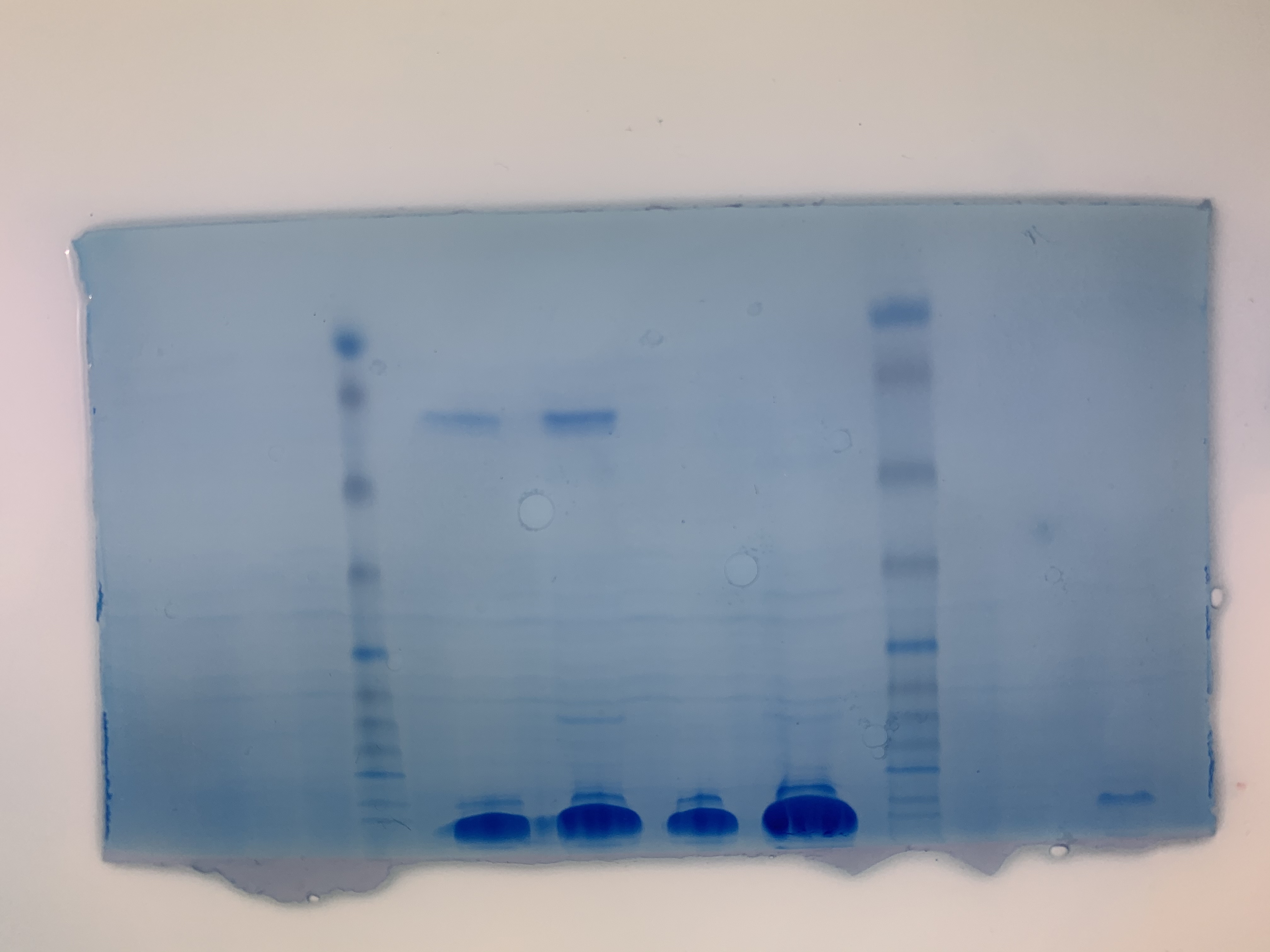

Supplement: Supplementary file 4 — source data [file 41467_2021_24458_MOESM4_ESM.zip › source data/Figure 1a.jpg]

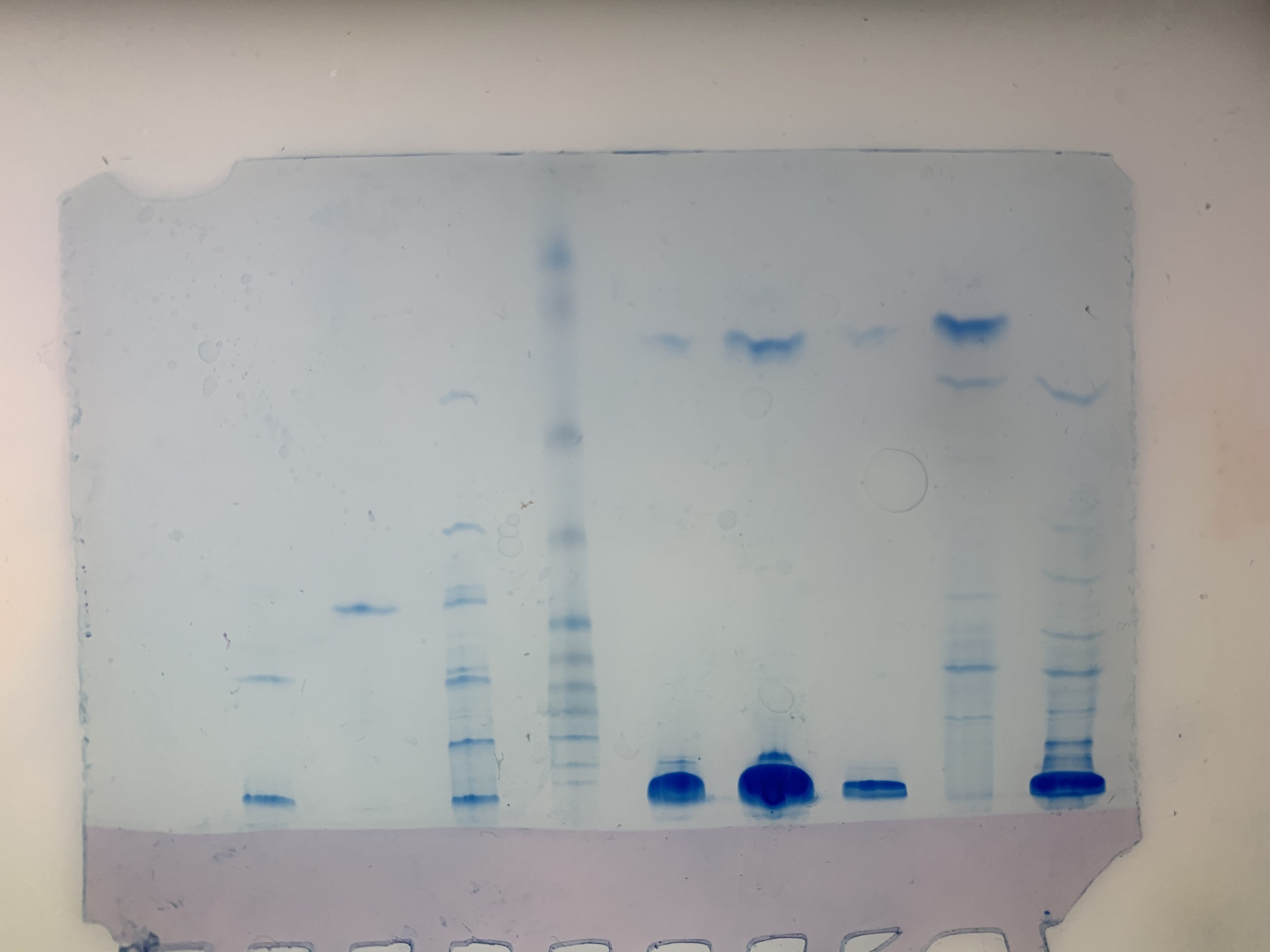

Supplement: Supplementary file 4 — source data [file 41467_2021_24458_MOESM4_ESM.zip › source data/Fingure 1b.jpg]

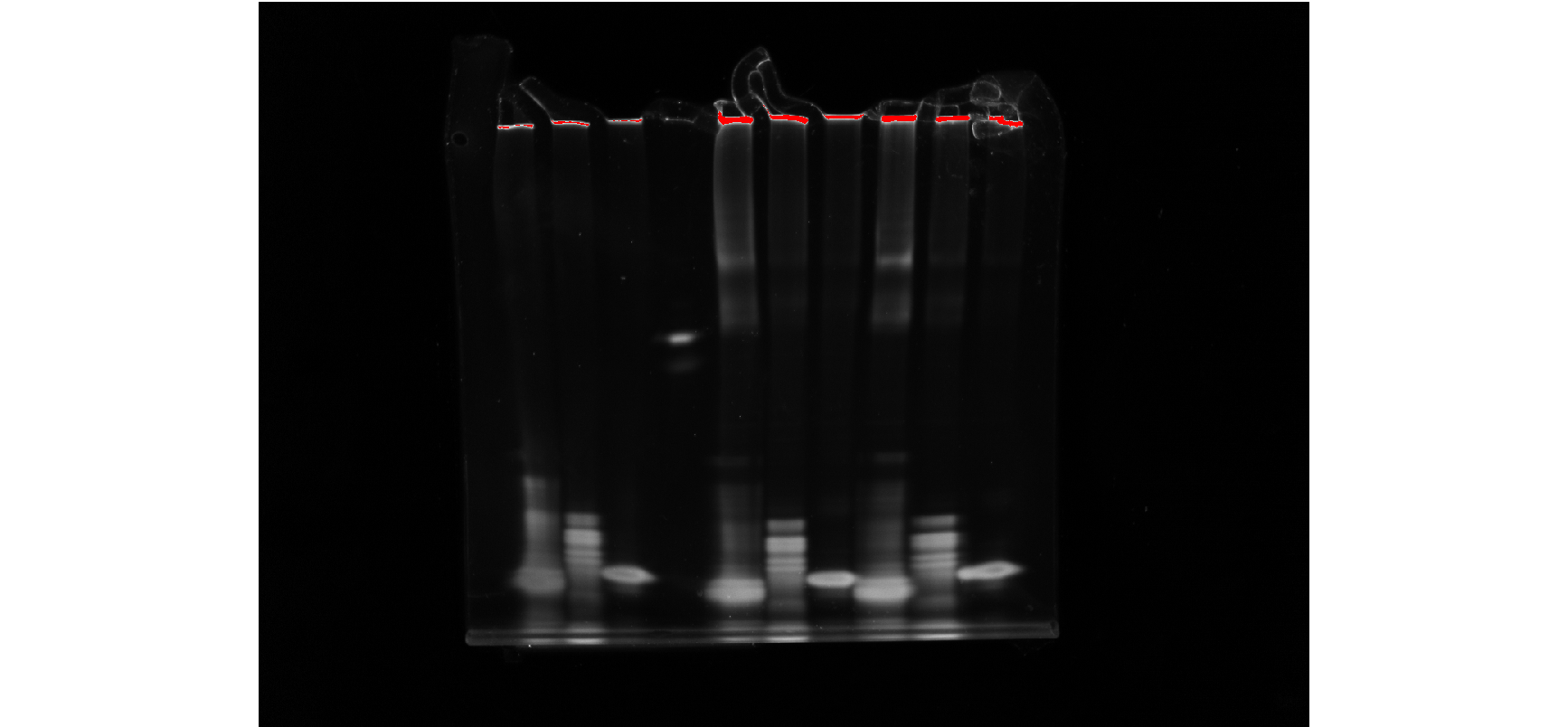

Supplement: Supplementary file 4 — source data [file 41467_2021_24458_MOESM4_ESM.zip › source data/Supplementary Figure 4 c.tif]
